# Overexpression of COL11A1 confers tamoxifen resistance in breast cancer

**DOI:** 10.1038/s41523-024-00645-3

**Published:** 2024-05-28

**Authors:** Chengxiao Fu, Shan Duan, Xiaoming Zhou, Yingcai Meng, Xisha Chen

**Affiliations:** 1 Cancer Research Institute, the First Affiliated Hospital, Hengyang Medical School, University of South China, Hengyang, Hunan China; 2 Department of Pharmacy, the First Affiliated Hospital, Hengyang Medical School, University of South China, Hengyang, Hunan China; 3 Department of Pulmonary and Critical Care Medicine, the First Affiliated Hospital, Hengyang Medical School, University of South China, Hengyang, Hunan China; 4 Institute of Drug Clinical Trial, the First Affiliated Hospital, Hengyang Medical School, University of South China, Hengyang, Hunan China; 5 Department of Pharmacy, Xiangya Hospital, Central South University, Changsha, Hunan China

**Keywords:** Breast cancer, Breast cancer

## Abstract

Breast cancer is the most commonly diagnosed malignancy and benefits from endocrine agents such as tamoxifen. However, the development of drug resistance in cancerous cells often leads to recurrence, thus limiting the therapeutic benefit. Identification of potential biomarkers that can predict response to tamoxifen and recognize patients who will clinically benefit from this therapy is urgently needed. In this study, we report that high collagen type XI alpha 1 (COL11A1) expression was associated with poor therapeutic response and prognosis in breast cancer patients treated with tamoxifen. To confirm the role of COL11A1 in the development of tamoxifen resistance, we established MCF-7/COL11A1 and T47D/COL11A1 cell lines, which stably expressed COL11A1. Compared with parental MCF-7 and T47D, MCF-7/COL11A1 and T47D/COL11A1 cells were more resistant to 4-OHT-induced growth inhibition. Moreover, the level of COL11A1 expression was upregulated in tamoxifen-resistant MCF-7/TamR and T47D/TamR cell lines, and depletion of COL11A1 markedly sensitized the cells to 4-OHT in vitro and in vivo. Interestingly, the level of estrogen receptor α (ERα) expression was elevated, probably due to the increased COL11A1 in TamR cells. In addition, knockdown of COL11A1 decreased the expression of ERα and its downstream target genes. Overall, our findings suggest that overexpressed COL11A1 contributes to tamoxifen resistance, and targeting COL11A1 holds great promise for reversing endocrine resistance.

## Introduction

Breast cancer has come to be the most commonly diagnosed cancer worldwide and remains the leading cause of cancer death in women^1^. Approximately 70% of breast cancers present as ERα positive (+) and respond to anti-estrogen therapy such as tamoxifen, a commonly prescribed endocrine agent that exerts anti-cancer effect via competing with estrogen for binding to ERα^2^. Despite the markedly improvement in survival rates of breast cancer patients, the development of drug resistance presents a major challenge in the clinic, for which severely limited the efficacy of endocrine therapy, leading to breast cancer recurrence and mortality^3^.

Thus far, there have been accumulating studies focused on elucidating the mechanisms underlying the tamoxifen resistance and several major pathways have been recognized to be involved in this process. Altered or aberrant ER transcriptional activity is one of the mechanisms that mediate tamoxifen resistance and promote tumor progression^4,5^. On the other hand, activation of receptor tyrosine kinases has been observed in endocrine resistant tumors and been reported to induce tamoxifen resistance^6–9^. In addition, cross-talk between alternative signaling pathways involving cell proliferation and survival has also been demonstrated to promote tamoxifen resistance^10–12^. In view of the complexity of mechanisms underlie resistance, it is imperative to identify potential molecular markers associated with therapeutic response.

Collagen type XI alpha 1 (COL11A1), one of three alpha chains of type XI collagen, belongs to the collagen family, the main component of extracellular matrix (ECM). Although it was initially reported to regulate bone development and collagen fiber assembly^13^, recent studies have recognized COL11A1 plays a crucial role in multiple types of cancer^14,15^. COL11A1 is frequently upregulated and correlated with aggressive phenotype and poor prognosis in ovarian, colorectal and pancreatic cancers^16–18^. High level of COL11A1 promotes tumor cell proliferation, migration and metastasis^19^. Further research found that COL11A1 mediates resistance of chemotherapy and immunotherapy^20–25^. All the evidence suggests the importance of COL11A1 in cancer progression.

In this study, we identified COL11A1 is overexpressed in ER-positive breast cancer and promotes cells resistant to tamoxifen. Knockdown of COL11A1 significantly enhanced the sensitivity of tamoxifen in TamR (tamoxifen-resistant) cells both in vitro and in vivo. We further revealed that the reversal of tamoxifen resistance caused by COL11A1 knockdown may be associated with the inhibition of ERα pathway. The results of our study provide an understanding of the role of COL11A1 in breast cancer and facilitate the development of novel treatment strategies to overcome endocrine therapy resistance.

## Results

### COL11A1 is upregulated in ER-positive breast cancer and correlates with poor endocrine therapeutic benefits

**Fig. 1:**
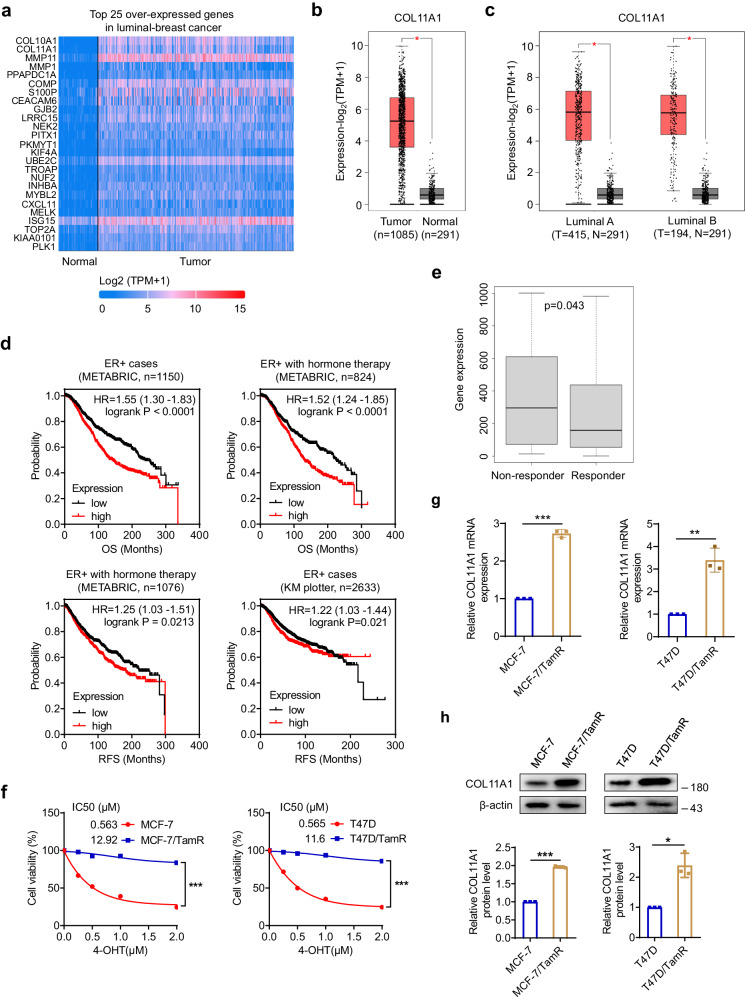
COL11A1 is upregulated in ER-positive breast cancer and correlates with poor endocrine therapeutic benefits. **a** The top 25 genes differentially expressed in luminal-breast cancer and normal tissues from UALCAN database. Heatmaps represent gene expression levels. **b** The GEPIA analysis of COL11A1 expression in breast cancer and normal tissues. **c** The GEPIA analysis of COL11A1 expression in luminal-breast cancer and normal tissues. The boxes represent the 25th to 75th percentile with the lines in the center showing the median. Whiskers extend from the minimum (Q1-1.5*IQR) to the maximum (Q3 + 1.5*IQR) value for each dataset. IQR, interquartile range. **d** Analysis of the effect of the COL11A1 expression level on overall survival (OS) and relapse free survival (RFS) of ER+ breast cancer patients with or without hormone therapy in the indicated cohorts from METABRIC and KM plotter online database. The data were assessed using the Kaplan-Meier method with the log-rank test. **e** The COL11A1 expressions in non-responder and responder groups in breast cancer cohorts from ROC plotter server. The boxes represent the 25th to 75th percentile with the lines in the center showing the median. Whiskers extend from the minimum to the maximum value for each dataset. **f** MCF-7, MCF-7/TamR, T47D, and T47D/TamR cells were treated by 4-OHT with indicated concentrations for 72 h, and cell viability was measured by CCK-8 assay. **g** The mRNA levels of COL11A1 in MCF-7, MCF-7/TamR, T47D, and T47D/TamR cells were evaluated using quantitative RT-PCR. **h** The protein expression levels of COL11A1 in MCF-7, MCF-7/TamR, T47D, and T47D/TamR cells were evaluated by western blotting and quantified. β-Actin was used as a loading control. Results shown are representative of three independent experiments. Data are represented as mean ± SD of biological triplicates. *p < 0.05; **p < 0.01; ***p < 0.001.

In view of the employment of endocrine therapy in treating ER-positive breast cancer, we analyzed the gene expression profiles of luminal-breast cancer and normal tissues from the publicly available online UALCAN database at first. Among the top 25 overexpressed genes in luminal-breast cancer, COL10A1 was the most highly elevated (Fig. 1a). For the purpose of trying to identify biomarkers for the prediction of endocrine therapy efficacy, we explored the association of COL10A1 expression and endocrine therapeutic response based on 5-year relapse-free survival in ER-positive breast cancer patients via ROC Plotter-Online ROC analysis database (https://www.rocplot.org/), and found that the expression of COL10A1 in responder and non-responder groups is equivalent (Supplementary Fig. 1). This result indicates COL10A1 may not be a treatment response-related gene and is not suitable for our further study; therefore, the next COL11A1 is regarded as a candidate for subsequent evaluation.

We first confirmed the expression of COL11A1 in breast cancer and normal breast tissue, and found COL11A1 showed higher level in breast cancer tissue compared to the normal breast tissue by GEPIA analysis (Fig. 1b). Consistently, the expression of COL11A1 was upregulated in luminal-breast cancer (Fig. 1c). We then examined the clinical relevance of COL11A1 in ER+ breast cancer cohort from the Molecular Taxonomy of Breast Cancer International Consortium (METABRIC)^26^. As shown in Fig. 1d, a high expression level of COL11A1 mRNA was associated with worse overall survival (OS) in ER+ breast cancers excluded cases died of other causes rather than tumor, as well as in patients who received hormone therapy; additionally, high COL11A1 expression showed shorter relapse-free survival (RFS) in hormone therapy-treated cases. A similar result was observed in ER+ patients from KM-plotter (Fig. 1d). We also evaluated the effect of COL11A1 on endocrine therapeutic responses of breast cancer patients by ROC plotter server, and demonstrated that COL11A1 expression was significantly elevated in the non-responder group compared to the responder group (Fig. 1e).

We then examined the expression level of COL11A1 between ER positive breast cancer cells (MCF-7 and T47D) and tamoxifen-resistant breast cancer cells (TamR) (Fig. 1f). Our results revealed that COL11A1 expression was significantly higher in TamR cells than that in wild type MCF-7 and T47D cells (Fig. 1g, h). Interestingly, these TamR cells with high COL11A1 expression also exhibited decreased response to fulvestrant, a selective estrogen receptor downregulator (Supplementary Fig. 2). Taken together, the above results indicate that COL11A1 may be involved in the regulation of the sensitivity of antiestrogen agent in breast cancer.

### Overexpression of COL11A1 inhibits breast cancer cell sensitivity to tamoxifen

**Fig. 2:**
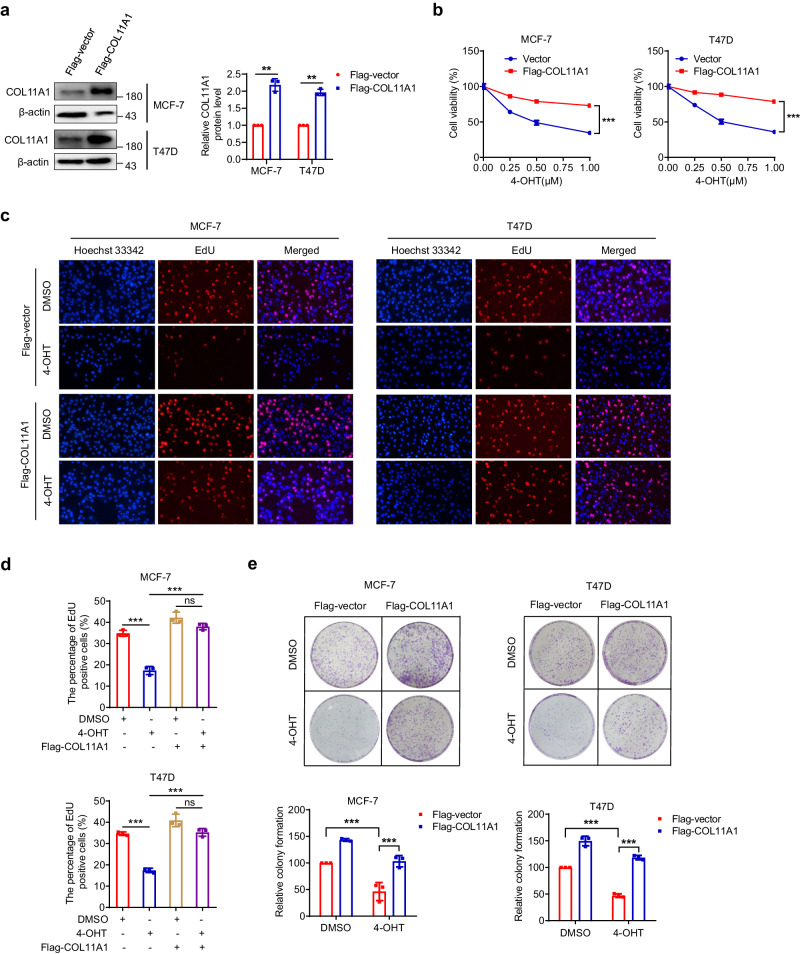
Overexpression of COL11A1 inhibits breast cancer cell sensitivity to tamoxifen. **a** MCF-7 and T47D cells were stably transfected with Flag-COL11A1 plasmids, and the overexpression efficacy was evaluated using western blotting by anti-COL11A1 antibody and quantified. β-Actin was used as a loading control. **b** MCF-7 and T47D cells stably expressing empty vector (EV) or flag-tagged COL11A1 were incubated with indicated concentrations of 4-OHT for 72 h, and cell viability was measured by CCK-8 assay. **c**, **d** Cell proliferation was measured using EdU after 72 h of incubation with 0.5 μM 4-OHT. Magnification, ×200. **e** Colony-formation assay of MCF-7 and T47D cells stably expressing empty vector (EV) or flag-tagged COL11A1 in the presence of DMSO or 0.5 μM 4-OHT. Results shown are representative of three independent experiments. Data are represented as mean ± SD of biological triplicates. *p < 0.05; **p < 0.01; ***p < 0.001. ns, no significance.

To investigate the role of COL11A1 in tamoxifen resistance, we stably transfected tamoxifen-sensitive, ER+ breast cancer MCF-7 and T47D cells with COL11A1 plasmid, and confirmed a notable increase of COL11A1 level (Fig. 2a). The cell viability of these cells in the absence and presence of 4-hydroxytamoxifen (4-OHT), the active metabolite of tamoxifen, was measured and compared. As shown in Fig. 2b, ectopically overexpression of COL11A1 dramatically attenuated the reduction in cell viability of breast cancer cells treated with increasing concentrations of tamoxifen (Fig. 2b). The numbers of cells positive for EdU staining were reduced in tamoxifen-treated control cells, while overexpression of COL11A1 blocked tamoxifen-induced decreases in EdU-positive cell numbers (Fig. 2c, d). Colony formation experiment further confirmed COL11A1 inhibited the sensitivity of breast cancer cells to tamoxifen (Fig. 2e). Collectively, these results suggest COL11A1 overexpression makes tamoxifen-sensitive breast cancer cells refractory to the drug. We also investigated the COL11A1 effect on the responsiveness of the cells to CDK4/6 inhibitors including palbociclib, abemaciclib and ribociclib, which are now frequently used in treating ER+ breast cancer patients^27–29^. Of note, overexpression of COL11A1 reduced the response of MCF-7 cells to the above three inhibitors (Supplementary Fig. 3), implying that high COL11A1 expression may induce multidrug resistance and it represents a promising therapeutic target.

### COL11A1 knockdown sensitizes TamR breast cancer cells to tamoxifen treatment in vitro

**Fig. 3:**
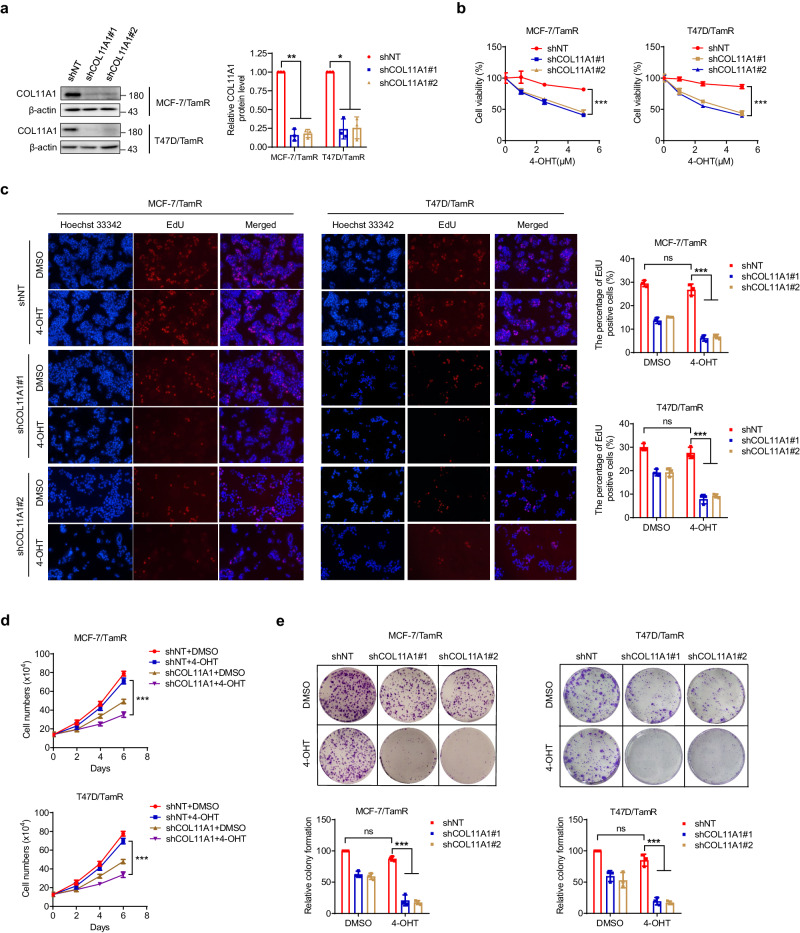
COL11A1 knockdown sensitizes TamR breast cancer cells to tamoxifen treatment in vitro. **a** The expressions of COL11A1 in MCF-7/TamR and T47D/TamR cells were knocked down by two independent shRNAs. **b** MCF-7/TamR and T47D/TamR cells with or without COL11A1 depletion were incubated with indicated concentrations of 4-OHT for 72 h, and cell viability was measured by CCK-8 assay. **c** MCF-7/TamR and T47D/TamR cells with or without COL11A1 depletion were incubated with 5 μM 4-OHT for 72 h, and cell proliferation was measured using EdU assay. **d** MCF-7/TamR and T47D/TamR cells with or without COL11A1 depletion were incubated with 5 μM 4-OHT for the indicated durations. Cell proliferation was analyzed by cell counting. **e** Colony-formation assay of MCF-7/TamR and T47D/TamR cells with or without COL11A1 depletion in the presence of DMSO or 5 μM 4-OHT. Results shown are representative of three independent experiments. Data are represented as mean ± SD of biological triplicates. *p < 0.05; **p < 0.01; ***p < 0.001. ns, no significance.

To further verify the above hypothesis, we determined to test whether inhibition of COL11A1 in TamR cells restores the sensitivity of tamoxifen. Firstly, we stably depleted COL11A1 using short hairpins in MCF-7/TamR and T47D/TamR cells, and observed that the COL11A1 protein level was reduced in COL11A1-knockdown cells (Fig. 3a). Our data demonstrated that in the shNT-TamR group, tamoxifen treatment did not lead to significant reduction in cell viability, while COL11A1 silencing dramatically increased tamoxifen-induced decreases in cell viability (Fig. 3b). We next selected 5 μM tamoxifen for subsequent experiments, and the result of EdU assay showed depletion of COL11A1 restored the inhibitory effect of tamoxifen on TamR cell proliferation (Fig. 3c). The re-sensitization effect of COL11A1 knockdown on tamoxifen was also confirmed by cell number counting and colony formation assays (Fig. 3d, e). These results indicate inhibition of COL11A1 is a hopeful approach to treating endocrine therapy–resistant breast cancer.

### COL11A1 enhances ERα signaling

**Fig. 4:**
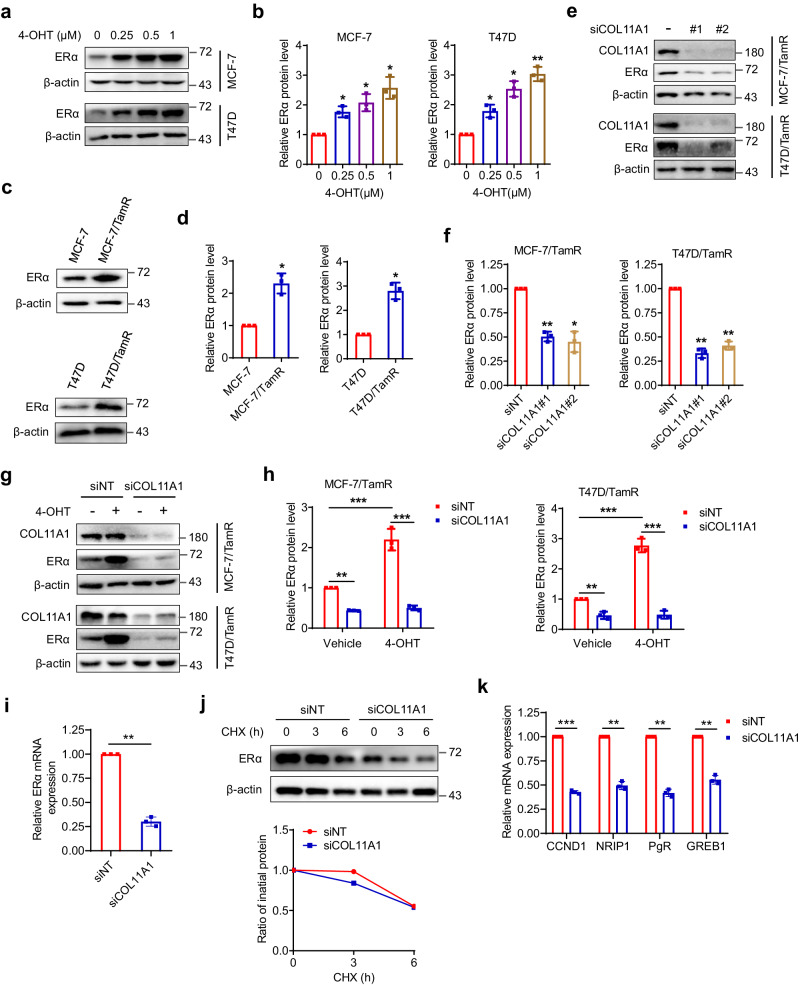
COL11A1 enhances ERα signaling. **a**, **b** Western blotting was used to detect the expression of COL11A1 in MCF-7 and T47D cells treated with indicated concentrations of 4-OHT for 72 h. **c**, **d** The protein expression levels of ERα in MCF-7, MCF-7/TamR, T47D, and T47D/TamR cells were evaluated by western blotting and quantified. **e**, **f** Western blotting analysis of ERα in MCF-7/TamR and T47D/TamR cells with or without COL11A1 knockdown. **g**, **h** MCF-7/TamR and T47D/TamR cells with or without COL11A1 knockdown were incubated with 5 μM 4-OHT for 72 h. The expression of ERα was examined by western blotting, β-Actin was used as a loading control. **i** qPCR analysis of the ERα mRNA expression in MCF-7/TamR cells with or without COL11A1 knockdown. **j** MCF-7/TamR cells transfected with the indicated siRNAs were treated with cycloheximide (CHX, 20 μg/ml), and collected at the indicated times for western blot. **k** qPCR analysis of the mRNA expressions of CCND1, NRIP1, PgR and GREB1 in MCF-7/TamR cells with or without COL11A1 knockdown. Results shown are representative of three independent experiments. Data are represented as mean ± SD of biological triplicates. *p < 0.05; **p < 0.01; ***p < 0.001.

It is known that crosstalk between ER and other signaling pathways under long-term exposure to TAM can reactivate ER and promote ER target gene expression, thus contributing to endocrine resistance^30,31^. We detected the expression changes of ERα upon stimulation of 4-OHT, and found that 4-OHT increased ERα protein levels in a dose-dependent manner in breast cancer cells (Fig. 4a, b). Importantly, compared to sensitive MCF-7 and T47D cells, we observed notable elevated ERα protein expression in TamR cells (Fig. 4c, d). The role of COL11A1 in controlling cell sensitivity to tamoxifen made us wonder whether ERα activity could be regulated by COL11A1. To this end, we examined the effect of COL11A1 modulation on ERα levels. As shown in Fig. 4e, f, COL11A1 knockdown remarkably down-regulated the protein level of ERα. However, the ERα protein expression was no longer stimulated in the presence of 4-OHT when COL11A1 was inhibited (Fig. 4g, h). Furthermore, COL11A1 silencing induced a significant decrease of ERα mRNA levels (Fig. 4i); whereas, knockdown of COL11A1 did not lead to obvious change in ERα protein stability (Fig. 4j), implying that ERα is modulated by COL11A1 at transcriptional level. To explicit the effect of COL11A1-regualted ERα on ER target gene expression, we carried out correlation analysis by the web server TIMER, and found that the expression of COL11A1 is positively correlated with CCND1 and NRIP1, two well-known ERα-target genes (Supplementary Fig. 4). We further performed RT-qPCR experiment and specified that COL11A1 silencing inhibited endogenous ERα-target gene expression including CCND1, NRIP1, PgR and GREB1 (Fig. 4k). Taken together, these data imply COL11A1 promotes endocrine resistance possibly through modulating ERα activity and downstream signaling.

### Knockdown of COL11A1 reverses tamoxifen resistance in TamR xenograft mouse model

**Fig. 5:**
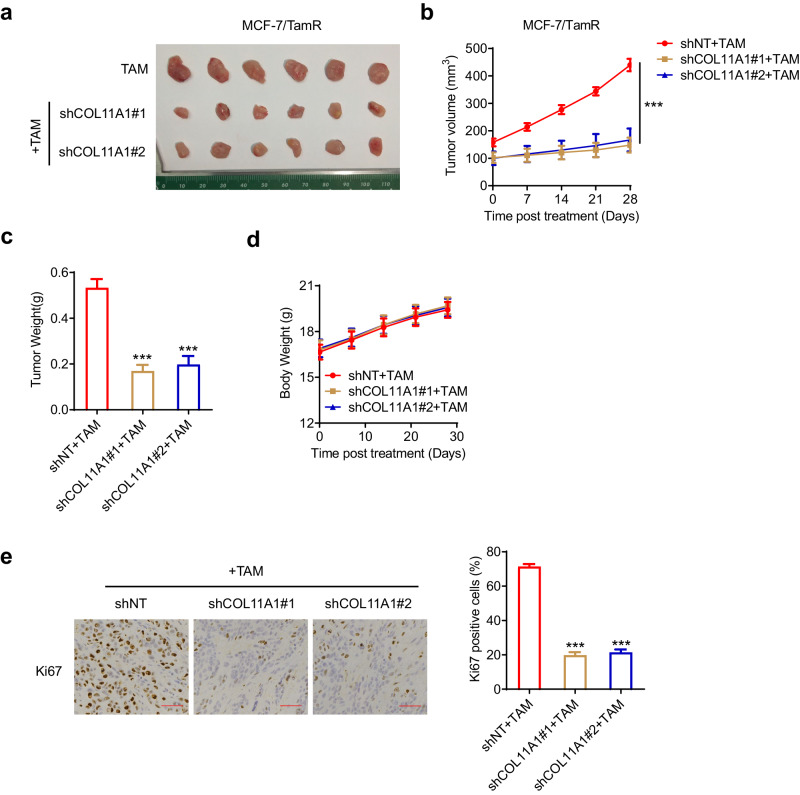
Knockdown of COL11A1 reverses tamoxifen resistance in vivo. 6-week-old female Balb/c nude mice were subcutaneously injected with shNT and shCOL11A1 transfected MCF-7/TamR cells and treated with tamoxifen (slow-release pellets). **a** Subcutaneous tumors were excised and photopraphs were taken at the termination of the experiment. **b** Tumor sizes were measured on the days as indicated. **c** Tumor weights were measured at the end of the experiments. **d** The effect of treatment on mice body weight. **e** Characterization of MCF-7/TamR xenograft tumors with histologic analysis by IHC staining of Ki67. Scale bar, 100 μm. All data represents the mean ± SD (*n* = 6). ***p < 0.001.

To investigate the translational potential of our findings, we evaluated the efficacy of the COL11A1 knockdown in combination with tamoxifen, in a TamR xenograft mouse model. Briefly, MCF-7/TamR cells with or without COL11A1 depletion were subcutaneously injected into the nude mice and then subjected to tamoxifen treatment. Compared to the control group, COL11A1 knockdown markedly retarded the growth of xenograft tumors throughout the entire treatment period, as evidenced by the reductions in tumor volume and tumor weight (Fig. 5a–c). No differences in mouse body weights were observed between control and COL11A1 depletion groups (Fig. 5d), indicating a negligible toxicity of this therapeutic regimen in tumor-bearing mice. In addition, the histopathologic characterization of the xenograft tumors demonstrated a decreased Ki67 staining in tumors from shCOL11A1 and tamoxifen co-treatment groups, implying a lessened proliferation rate (Fig. 5e). Together, these observations suggest COL11A1 serves as a promising therapeutic target for TamR breast cancer and inhibition of COL11A1 potentially leads to restoration of sensitivity to tamoxifen.

## Discussion

Estrogen receptor targeted therapy via using tamoxifen and other endocrine agents is highly effective in blocking tumor growth in ER-positive breast cancers. In spite of the initial achievements in reducing disease mortality and prolonging survival, long term endocrine treatment frequently leads to the development of drug resistance, with the fact that cancer recurs in about one-third of patients treated with tamoxifen^32,33^. Hence, it has become urgent to reveal mechanisms and molecules responsible for acquisition of tamoxifen resistance and to exploit combination therapies to improve treatment for breast cancer. Increasing efforts have been made to try to advance this issue^34^. In this study, we demonstrated COL11A1 confers tamoxifen resistance and represents a promising therapeutic target in breast cancer.

COL11A1 was previously identified as a specific marker for cancer-associated fibroblasts (CAFs) and primarily overexpressed by a subset of CAFs adjacent to cancer cells or within the tumor. In addition, when co-cultured with cancer cells, normal fibroblasts could be stimulated to upregulate COL11A1, and finally facilitate malignant progression^35^. Accumulating evidence further indicate that COL11A1 mediates the interplay between different cell types in the TME to accelerate malignancy^36–38^. A variety of reports have demonstrated a remarkable increase in the expression of COL11A1 in various cancer types, such as pancreatic, ovarian, lung, esophageal, colorectal, gastric and breast cancers^39–41^. And more importantly, overexpression of COL11A1 closely correlated with poor prognosis in human malignant tumors. Previous studies have showed that COL11A1 induces chemoresistance in epithelial ovarian cancer and non-small cell lung cancer. According to two recent studies, COL11A1 also regulates immune infiltration in breast cancer, suggesting COL11A1 plays an important role in tumor immunity^42,43^.

Here, to our knowledge, we showed for the first time that COL11A1 is highly upregulated in breast cancer cells that are resistant to tamoxifen. We also found that elevated COL11A1 indicates insensitivity to tamoxifen and poor prognosis in breast cancer patients. We further conducted experiments and verified that exogenously overexpression of COL11A1 promotes breast cancer cells resistant to tamoxifen, while COL11A1 knockdown restores sensitivity of TamR cells to tamoxifen both in vitro and in vivo. Previously, it was reported that in acquired tamoxifen resistance, ER is still retained detectable levels and continues to promote cell proliferation in most tumors, indicating that activated ER signaling can still serve as a therapeutic target. We demonstrated herein that the level of ERα is elevated in TamR breast cancer cells. Notably, we were surprised to find that the cells with high COL11A1 that are resistant to tamoxifen even become less sensitive to fulvestrant to some extent when compared with the parental MCF-7 and T47D cells, and this may be attributed to the compensatory upregulation of ERα expression in COL11A1-overexpressed TamR cells. Moreover, we observed that knockdown of COL11A1 reduces ERα expression both in mRNA and protein level, but has little effect on ERα protein stability. Thus, these results support a point that the increased ERα expression in tamoxifen-resistant breast cancer is probably the result of enhanced COL11A1 levels. Certainly, further research is necessary to clarify the precise mechanism by which COL11A1 transcriptionally regulates ERα.

In view of that ERα promotes gene transcription via directly binding to an ERE in the target gene or by interacting with other transcription factors such as AP-1 complex, we wondered whether COL11A1-modulated ERα is functionally affected. To test this hypothesis, we transfected cells with COL11A1 siRNA and examined ERα target genes expression with or without EREs in the promoter region. As expected, COL11A1 knockdown decreased the mRNA expression of PgR, GREB1 (with ERE), CCND1 and NRIP1 (without ERE), indicating ERα-mediated transcriptional function is disrupted. In our extended experiment, we found that high level of COL11A1 contributed to reduced sensitivity of CDK4/6 inhibitors in ER+ breast cancer cells, suggesting inhibition of COL11A1 may enhance the efficacy of palbociclib, abemaciclib and ribociclib. So, the exploitation of COL11A1 inhibitors will be of great significance. In addition, it is worthwhile to reveal upstream regulators of COL11A1 to help identify novel potential therapeutic targets.

## Methods

### Cell lines and culture

MCF-7, T47D, MCF-7/TamR, and T47D/TamR cells were used. The human breast cancer cell MCF-7 was acquired from Cell Resource Center of Institute of Basic Medical Sciences of CAMS/PUMC, Beijing, China. T47D cell was purchased from Cell Bank of Chinese Academy of Sciences, Shanghai, China. Cell lines were authenticated using STR profile analysis and periodically checked to confirm they were mycoplasma free. MCF-7/TamR and T47D/TamR cells were induced as an in vitro acquired tamoxifen resistance model via exposing MCF-7 and T47D cells to long-term 1 μM 4-OHT treatment^44^. MCF-7 and MCF-7/TamR cells were cultured in DMEM medium. T47D and T47D/TamR cells were maintained in RPMI 1640. Both tamoxifen-resistant cells were cultured in medium containing 1 μM 4-OHT to maintain the resistance. All cell culture media were supplemented with 10% fetal bovine serum, 100 units/mL penicillin and 100 μg/mL streptomycin. All cell lines were maintained at 37 °C in a 5% CO_2_ incubator.

### Antibodies and Reagents

Antibodies used in immunoblotting: COL11A1 (ab64883, Abcam, 1:1000 dilution), ERα (No.8644, CST, 1:1000 dilution), anti-β-actin (60008-1-lg, Proteintech, 1:5000 dilution). Normal IgG/Peroxidase-conjugated AffiniPure Goat Anti-Rabbit/Mouse IgG (H + L) was purchased from Jackson Immuno Research. Tamoxifen metabolite 4-hydroxytamoxifen (4-OHT) was purchased from Sigma. Palbociclib HCl (#S1116), abemaciclib mesylate (#S7158), ribociclib HCl (#S5187) and fulvestrant (#S1191) were obtained from Selleck (Shanghai, China).

### siRNA, shRNA and plasmid transfection

siRNAs targeting COL11A1 was designed and synthesized by RiboBio (Guangzhou, China). Transfection of siRNA was carried out according to the manufacturer’s protocol. Stable clones were selected and maintained in 1 μg/mL of puromycin (Sigma).

### Western blot analysis

Cells were lysed at ice for 30 min in RIPA supplemented with a protease inhibitor cocktail (Biotool), followed by centrifugation at 12,000 × *g* for 15 min. Proteins (15–30 μg) were resolved by SDS-PAGE and then transferred to PVDF membrane. After blocking with skim milk, the PVDF membranes were incubated with the respective antibodies in 5% BSA at 4 °C overnight, followed by incubation of a secondary antibody at room temperature for 1 h. The protein signals were detected by ECL method. All blots derive from the same experiment and were processed in parallel. Original images of uncropped western blot membranes are shown in Supplementary Fig. 5.

### Pulse-chase assay

To measure the effect of COL11A1 on ERα protein stability, the MCF-7/TamR cells transfected with the indicated siRNAs were treated with the protein synthesis inhibitor cycloheximide (CHX, #S7418, Selleck) for the indicated durations before collection, and then subjected to western blot analysis.

### Quantitative real-time PCR

Total RNA from cell lines was isolated using the Trizol reagent (Biotech) and 1st strand cDNA was synthesized using PrimeScript RT Reagent Kit (Perfect real time) (Takara). Real time PCR was performed using SYBR Premix Ex Tap (Tli RNaseH Plus) (Takara), and was run on Bio-Rad. For quantification of gene expression, the 2^−ΔΔCt^ method was used. GAPDH expression was used for normalization.

### Cellular viability assay

Cells plated at 5 × 10^3^ cells per well in 96-well tissue culture plates were subjected to indicated treatments, and then incubated at 37 °C for indicated time in a humidified atmosphere containing 5% CO_2_/95% air. At the end of treatment, cell viability was measured with CCK8 (Bimake, Shanghai, China) reagent, and the OD value at 450 nm was detected to determine cell viability.

### Clonogenic assay

Cells were plated in 6-well tissue culture plates (500 cells per well) and treated with 4-OHT. After the incubation of about 15 days at 37 °C in a humidified atmosphere containing 5% CO_2_/95% air, cells were fixed with 4% paraformaldehyde and stained with crystal violet for 20 min, washed with PBS, and then the colonies were counted.

### 5-Ethynyl-2′-deoxyuridine assay

The cells were incubated with 5-Ethynyl-2′-deoxyuridine assay (EdU; Ribobio) for 2 h, and processed according to the manufacturer’s instruction. After three washes with PBS, the cells were incubated with 100 μL of 1× Apollo reaction cocktail for 30 min. Then cells were washed three times with 0.5% Triton X-100. The DNA contents were stained with 100 μL of 1× Hoechst 33342 (5 μg/mL) for 30 min and visualized under a fluorescence microscope.

### Bioinformatics analysis

The UALCAN cancer database was used to identify overexpressed genes in luminal-breast cancer. GEPIA 2 (Gene Expression Profiling Interactive Analysis 2) was used to compare COL11A1 expression between tumor and normal tissues of breast cancer samples. The prognostic value of COL11A1 expression was evaluated using the Molecular Taxonomy of Breast Cancer International Consortium (METABRIC) and Kaplan-Meier Plotter (www.kmplot.com) which contained gene expression data and survival information of clinical breast cancer patients. To analyze the association between COL11A1 expression level and OS/RFS, patient samples were divided into two groups (high vs low expression) according to COL11A1 mRNA expression (METABRIC, median; KM-plotter, upper quartile) and assessed using the Kaplan-Meier method, with the hazard ratio (HR) with 95% confidence intervals (CI) and log rank p value. ROC plotter server was utilized to analyze the correlation between the expression of COL11A1 and the therapeutic responses of BC patients to endocrine therapy. The correlation between COL11A1 and CCND1, NRIP1 expression was evaluated using the Gene_Corr module of TIMER (timer. cistrome.org) web resource^45^.

### In vivo studies

Animal studies were approved by the Animal Care and Use Committee of the First Affiliated Hospital of University of South China. Briefly, 2 × 10^6^ MCF-7/TamR cells mixed with equal volume of Matrigel were injected subcutaneously into 6-week-old female nude mice. Estrogen pellets (Innovative Research, 60-d slow-release pellet containing 0.72 mg) were implanted on the same day of cell inoculation. Tumor volume was calculated as length × width^2^× (π/ 6). When the tumor size reached about 100 mm^3^, six mice in each group were treated with tamoxifen pellet implants s.c. (5 mg, 60-day release). Tumor sizes and body weights were measured as indicated. After 4 weeks of treatment, mice were anaesthetized and then euthanized by cervical dislocation and tumors were sectioned and formalin-fixed and paraffin-embedded and IHC stained slides were made.

### Statistical analysis

All experiments were performed at least three times. The results are shown as mean ± SD of biological triplicates. Comparison between two groups was analyzed using two-tailed Student’s t-test. Comparison of multiple groups (>2) were performed using one-way analysis of variance. The Kaplan-Meier method and Gehan-Breslow-Wilcoxon test were used for the survival data analysis. GraphPad Prism V.6.01 was used to perform statistical analysis. *P* value < 0.05 was considered statistically significant.

### Supplementary information

Supplementary figures

nr-reporting-summary

## Data Availability

Data are available from the corresponding author upon reasonable request.

## Code availability

The bioinformatics analysis used in this work was conducted using publicly available datasets and was described in the “Methods” section.
