# Supplementary material for: Overexpression of COL11A1 confers tamoxifen resistance in breast cancer
Source: NPJ Breast Cancer. 2024 May 28;10:38. doi: 10.1038/s41523-024-00645-3 (PMC11133424; doi:10.1038/s41523-024-00645-3)
Supplement: Supplementary file 1 — Supplementary figures [file 41523_2024_645_MOESM1_ESM.pdf]

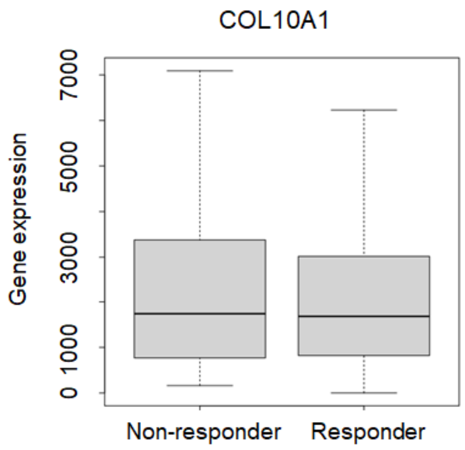

Supplementary figure 1. The expression of COL10A1 is equivalent in responder and non-responder groups. COL10A1 expressions in responder and non-responder groups of breast cancer cohorts generated by ROC Plotter-Online tool. The boxes represent the 25th to 75th percentile with the lines in the center showing the median. Whiskers extend from the minimum to the maximum value for each dataset.

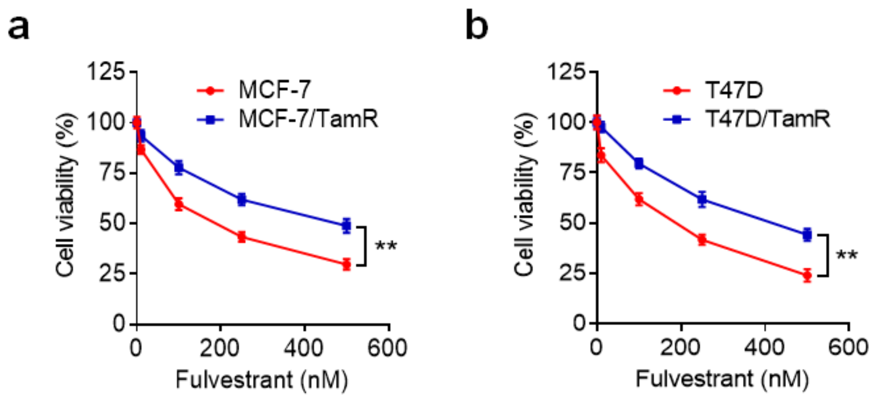

Supplementary figure 2. COL11A1-overexpressed TamR breast cancer cells is less sensitive to fulvestrant. MCF-7, MCF-7/TamR, T47D, and T47D/TamR cells were treated with 0nM, 10nM, 100nM, 250nM and 500nM fulvestrant for 72h, and cell viability was measured by CCK-8 assay. Data are represented as mean ± SD of biological triplicates. **, p<0.01.

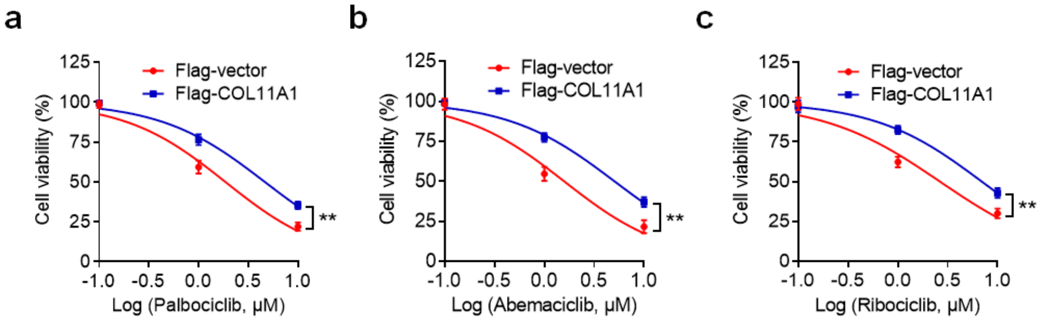

Supplementary figure 3. Overexpression of COL11A1 reduces the sensitivity of MCF-7 cells to CDK4/6 inhibitors. MCF-7 cells transfected with the indicated plasmids were treated with palbociclib (a), abemaciclib (b) and ribociclib (c) for 72h, and cell viability was measured by CCK-8 assay. Data are represented as mean ± SD of biological triplicates. **, p<0.01.

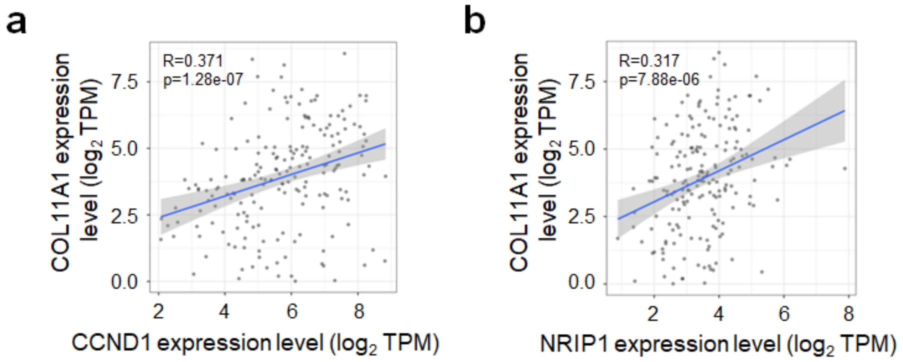

Supplementary figure 4. COL11A1 is positively correlated with ERα target genes expression. Analysis of the correlation between COL11A1 expression and CCND1, NRIP1 in BRCA using TIMER database Scatter plot of correlations.

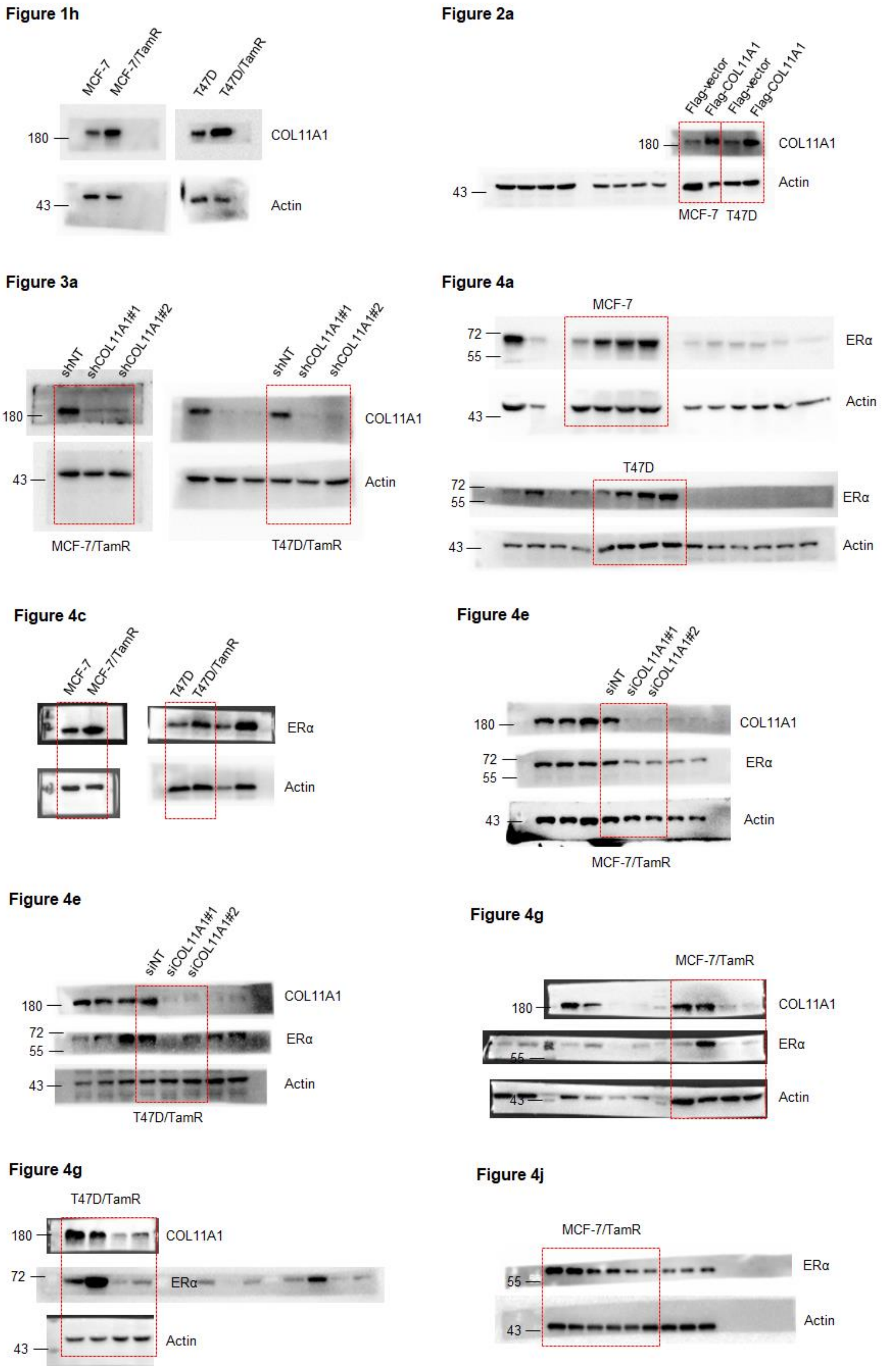

Supplementary figure 5. Uncropped western blot images from indicated figures.
